# Gender differences in sun protection use and beliefs amongst outdoor athletes

**DOI:** 10.1097/JW9.0000000000000221

**Published:** 2025-11-06

**Authors:** Gabrielle Keller Goff, Olivia A. Paraschos, Arlene Ruiz de Luzuriaga

**Affiliations:** a Pritzker School of Medicine, University of Chicago, Chicago, Illinois; b Department of Medicine, Section of Dermatology, University of Chicago, Chicago, Illinois

**Keywords:** sunscreen, athletes, female, skin cancer, melanoma, outdoor

**What is known about this subject in regard to women and their families?**

- Athletes who play outdoor sports receive large, frequent doses of ultraviolet radiation, which can increase their risk of developing skin cancer.
- Prior research has shown low rates of sun protection use amongst outdoor athletes, with few studies focusing on female athletes (FA) or sex differences.

**What is new from this article as messages for women and their families?**

- Despite higher rates of sunscreen use, FA reported sunburns at higher rates than male athletes.
- Blistering sunburns were common amongst outdoor athletes, with nearly a third of FA having been affected.
- FA were more likely to be deterred from using sunscreen by irritation to the skin than male athletes.

## Introduction

Despite women’s sports making major advances over the past several decades, research focusing on the health of female athletes (FA) or comparing differences between the genders has significantly lagged behind.^1,2^ A 2023 study found that only 8.8% of literature from leading sports medicine journals focused on FA, compared to 70.7% focusing on male athletes (MA). 20.5% included both males and females.^1^ Sun exposure is a pertinent health concern for athletes who play outdoor sports due to prolonged ultraviolet radiation exposure. This potentially increases the risk of developing skin cancer, especially with inconsistent use of sun protection.^3^ Despite this risk, relatively few studies have focused on sun protection amongst outdoor athletes, and even fewer have focused on FA or gender differences.^4,5^ These limited studies have found that FA may be more likely to engage in sun protective behaviors than males.^4,5^ To investigate further this issue, we administered a survey evaluating sun exposure, use of sun protection, and attitudes towards sun protection.

## Materials and methods

One hundred thirty-five adult outdoor athletes were recruited anonymously via Prolific Academic (estimated survey response rate 40–50%, https://www.prolific.com/) and were directed to an anonymous Qualtrics survey. All participants signed a consent form and were compensated. The University of Chicago Institutional Review Board provided oversight. The survey included questions in the following areas: demographic questions (eg, race, age, and gender), history of playing outdoor sports, assessment of behaviors around sun protection, and assessment of attitudes and beliefs towards sun protection. Statistical analysis was performed using Microsoft Excel.

## Results

Fifty-six participants (41.5%) were FA, 5 (3.7%) were nonbinary or preferred not to specify, and 74 (54.8%) were MA. The participants had the following distribution of self-reported Fitzpatrick skin types: 5.2% (type I), 17.8% (type II), 37.0% (type III), 24.4% (type IV), 10.4% (type V), 5.2% (type VI). The median age was 33 (range 18–64). 73 (54.1%) athletes played their sport at a recreational or intramural level, and 62 (45.9%) played their sport at a club, collegiate, or professional level.

**Fig. 1.**
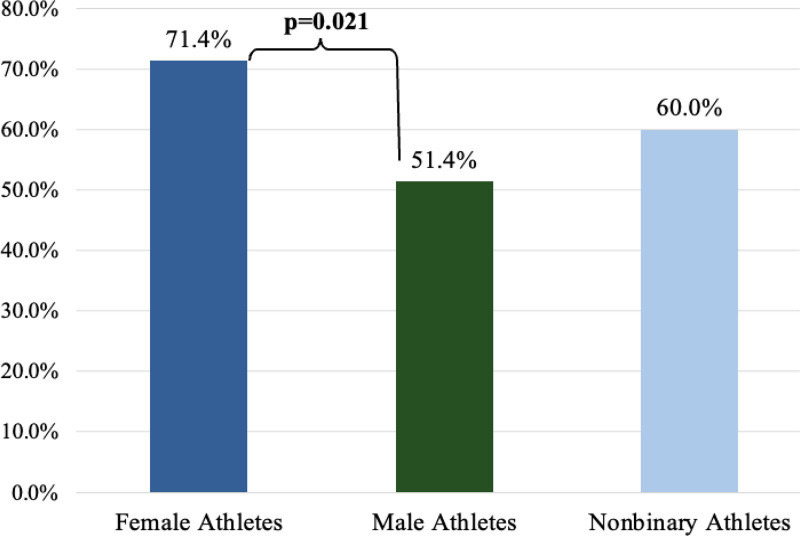
Gender differences in sunburns over the past 6 months. FAs were more likely than MAs to report that burning or stinging caused them to not wear sunscreen (21.4 vs 8.1%, *P* = .03) (Fig. 2). FA, female athletes; MA, male athletes.

FAs were more likely to report recent sunburns than MAs (71.4 vs 51.4%, *P* = .02) (Fig. 1).

## Discussion

While FAs used sunscreen more consistently than MAs, FAs reported higher rates of sunburns. Reasons for this may include improper sunscreen application, apparel differences, and differences in sports played.^6^ Limitations include sample size, possible recall and sampling biases, and an underestimation of response rate.

Repeated sunburns put athletes at increased risk for skin cancer. Proper counseling on adequate sun protection for these patients can help decrease this risk and keep FA healthy and active. Future studies may focus on gender differences in various countries, the underlying causes for gender differences, decreasing irritation from sunscreen, and interventions to increase proper sunscreen usage for outdoor athletes.

**Fig. 2.**
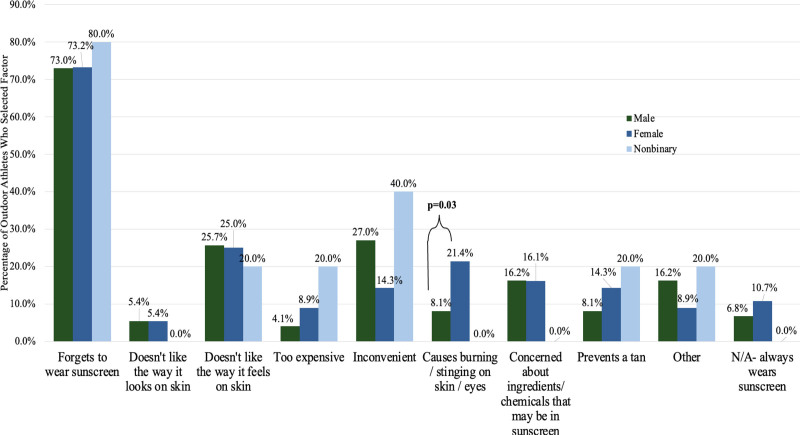
Gender differences in sunscreen deterring factors. FAs reported wearing sunscreen at higher rates than MAs when playing outdoor sports (55.4 vs 35.1%, *P* = .02). None (0/5) of NAs reported wearing sunscreen consistently. 32.1% of FAs, 33.8% of MAs, and 80.0% of NAs reported a history of blistering sunburns. FA, female athletes; MA, male athletes; NA, nonbinary/preferred not to specify athletes.

## Conflicts of interest

None.

## Funding

The authors would like to thank the University of Chicago Pritzker School of Medicine for their support of this research.

## Study approval

This study was approved by the Institutional Review Board of the University of Chicago and conducted in accordance with Institutional Review Board policies.

## Author contributions

GKG: Research design, writing of manuscript, performance of research, and data analysis. OAP: Writing of manuscript and data analysis. ARL: Research design, writing of manuscript, and data analysis.

## Patient consent

Informed, written consent was obtained for all study participants.

## Acknowledgments

The authors would like to thank the University of Chicago Pritzker School of Medicine for their support of this research.
